# Survival and complications in older adult patients with resectable gastric cancer according to number of resected lymph nodes: a cohort study

**DOI:** 10.1186/s12876-026-04703-x

**Published:** 2026-02-24

**Authors:** Camilo Ramírez-Giraldo, Isabella Van-Londoño, Maria Victoria Brina, Juliana Bueno-Marin, Camilo Bedoya-Motta, Edgar Javier Aguirre-Salamanca, Andrés Isaza-Restrepo

**Affiliations:** 1 Hospital Universitario Mayor - Méderi, Bogotá, Colombia; 2 Universidad del Rosario, Bogotá, Colombia

**Keywords:** Gastric cancer, Lymphadenectomy, Lymph node dissection, Older adults, Elderly, Prognosis

## Abstract

**Background:**

Gastric cancer (GC) incidence in older adults is usually higher than in the general population. Whereas surgical resection accompanied by an extended lymphadenectomy is the current standard treatment for GC, the impact of the extent of lymphadenectomy on survival in older adult patients has not been sufficiently studied and may be associated with a higher rate of complications in this group of patients.

**Materials and methods:**

An observational retrospective cohort study was performed in patients aged ≥ 75 years with a diagnosis of GC who underwent gastrectomy with curative intent to evaluate the influence of the number of retrieved lymph nodes (< 25 vs. ≥25) on postoperative morbidity and mortality and overall survival (OS).

**Results:**

A total of 122 patients were included in this study; 64 were included in the group with ≥ 25 retrieved lymph nodes and 58 in the group with < 25 retrieved nodes. Patients were predominantly male (61.5%) with a median age of 79.00 (IQR: 77.00–81.00) years. The lymph node ratio was an independent risk factor for OS (HR, 8.79; 95% CI, 2.35–32.85; *p* = 0.001), whereas the number of retrieved lymph nodes was not associated with differences in OS and was not identified as an independent risk factor for major postoperative complications.

**Conclusion:**

We did not identify that a higher number of retrieved lymph nodes was associated with an improvement in overall survival in patients aged ≥ 75 years; however, we observed a high rate of major postoperative complications in this population. Surgical decision-making in older patients with GC should be individualized, and the risk–benefit ratio must be carefully considered.

## Introduction

Global incidence and mortality associated with cancer increase rapidly with age as a consequence of population growth, increased life expectancy, and changes in the distribution of risk factors. According to GLOBOCAN, in 2020 gastric cancer (GC) ranked fifth among malignant disease diagnoses and fourth as a cause of cancer-related death worldwide [1]. While the crude incidence rate for all age groups is 12.3 per 100,000, this increases to 89.1 per 100,000 in populations older than 70 years [2, 3]. This increase warrants special attention to all aspects associated with this disease in older adult patients.

The current cornerstone of treatment for patients with GC with curative intent is radical gastrectomy (R0), complemented by different oncological therapy schemes developed over the last decades [4–6]. Considering that the primary route of malignant dissemination in GC is through lymphatic metastasis to regional lymph nodes [7], radical surgery implies an extensive lymphadenectomy; as such, the number of retrieved lymph nodes represents one of the main factors associated with prognosis. Accordingly, most clinical practice guidelines recommend performing extended (D2) lymphadenectomy for survival improvement [3, 8–12]. However, improved survival associated with extended lymphadenectomy has only been demonstrated after more than 15 years of follow-up [13, 14], a finding that justifies special consideration when applying this recommendation in older adult patients.

Additionally, age has been identified as an independent risk factor for survival [13–15]. Because of the increased risk of postoperative complications, mortality, higher comorbidity burden from causes other than the primary tumor, greater frailty, and reduced life expectancy, older adult patients have frequently been excluded or underrepresented in clinical trials and other studies that inform most treatment recommendations [3, 16, 17]. For similar reasons, chemotherapy regimens are often contraindicated or discontinued prematurely, rendering the surgical procedure a key determinant of short- and long-term outcomes [18]. GC treatment in older adult patients therefore remains a controversial topic in the current literature [19].

Considering all these factors, and the United Nations’ call to strengthen research and programs focused on the older population [20], this study was designed to evaluate surgical and oncological outcomes in patients aged ≥ 75 years with resectable GC according to the number of retrieved lymph nodes (< 25 vs. ≥25) as part of comprehensive surgical management.

## Materials and methods

### Study design

We conducted a retrospective observational cohort study. Convenience sampling was performed. All patients aged ≥ 75 years who underwent gastrectomy with curative intent between 2014 and 2024 were included. Data on death and date of death were extracted from the National Database of the Social Security in Health General System Resources Administration (ADRES). In accordance with national regulations and institutional policies, informed consent for the use of clinical information for research purposes was obtained. This study was reviewed and approved by our institution’s Ethics Committee and Technical Research Committee (approval number DVO005 2598-CV1751). We followed the STROBE guidelines to report this study [21].

### Patients

The preoperative workup included physical examination, esophagogastroduodenoscopy with biopsies, and computed tomography of the chest, abdomen, and pelvis. Additional diagnostic studies or staging laparoscopy were selectively performed to define clinical tumor staging (cTNM) in cases in which routine imaging was inconclusive. Additionally, a nutritional assessment was performed.

Neoadjuvant or adjuvant treatment, as well as the extent of gastrectomy, were defined by a multidisciplinary team, considering intraoperative findings when appropriate. A complete exploration of the abdominal cavity was performed prior to resection. The surgical approach (open or minimally invasive), type of reconstruction, and lymphadenectomy were defined according to the attending surgeon’s criteria, taking into account the patient’s clinical condition and tumor pathology. The goal of surgical resection was to obtain an en bloc, margin-free resection.

We analyzed the following data: patient demographics, body mass index, American Society of Anesthesiologists (ASA) Physical Status classification; previous diagnoses of hypertension, diabetes mellitus, chronic obstructive pulmonary disease, and/or chronic kidney disease; Charlson comorbidity index; Eastern Cooperative Oncology Group (ECOG) performance status; Barthel index; frailty status; nutritional status (Mini Nutritional Assessment–Short Form); calf circumference; hemoglobin; serum albumin; tumor histology, stage, and location; chosen surgical technique and approach, including adjacent organ resections (e.g., in cases of neoplastic involvement of adjacent organs, an en bloc resection was performed when curative intent was feasible); resection margin (R0 resection was defined as complete macro- and microscopic tumor clearance, whereas R+ resection indicated incomplete oncological resection); number of retrieved lymph nodes; number of metastatic lymph nodes; number of retrieved nodes (< 25 or ≥ 25); lymph node ratio (calculated as the number of metastatic lymph nodes divided by the total number of retrieved lymph nodes); neoadjuvant treatment; adjuvant treatment; length of hospital stay; requirement for surgical reintervention; and major complications (Clavien–Dindo grade ≥ III).

We excluded patients with gastric tumors who met any of the following criteria: histological diagnoses other than adenocarcinoma, presence of distant metastases, surgical reports that did not document the number of retrieved lymph nodes, or medical records lacking information on perioperative morbidity or mortality.

### Statistical analyses

Qualitative variables were summarized using frequencies and percentages. Quantitative variables were described using measures of central tendency and dispersion, according to their distribution (mean and standard deviation or median and interquartile range). Normality was assessed using the Shapiro–Wilk test. Univariate analyses were performed using the chi-square test for categorical variables and the Mann–Whitney U test for continuous variables to compare differences between groups based on the number of retrieved lymph nodes. The cutoff of 25 retrieved lymph nodes was selected a priori based on previous studies in older patients, particularly those by Passot et al. and Brenkman et al., which used similar thresholds to define a high lymph node yield [22, 23].

To evaluate the influence of the number of retrieved nodes (< 25 vs. ≥25) on postoperative morbidity and mortality, univariable and multivariable logistic regression analyses were performed, reporting odds ratios (ORs) with 95% confidence intervals (95% CIs). For multivariable logistic regression analyses, both non-parsimonious and parsimonious models were constructed to select covariates.

Survival was estimated using the Kaplan–Meier method, including postoperative deaths (intention-to-treat analysis). Survival curves were compared using the log-rank test. The association between the number of retrieved lymph nodes (< 25 vs. ≥25) and overall survival (OS) was evaluated using univariable and multivariable Cox proportional hazards models, reporting hazard ratios (HRs) with 95% CIs. For multivariable Cox analyses, both non-parsimonious and parsimonious approaches were used for variable selection.

The proportional hazards assumption was assessed using Schoenfeld residuals. When time-varying coefficients were identified, the follow-up time was split into predefined intervals, and the Cox model was stratified accordingly. A two-sided p value < 0.05 was considered statistically significant. All analyses were performed using RStudio (version 2023.12.1 + 402) [24–26].

## Results

A total of 122 patients were included in this study. The median age was 79.00 years (interquartile range [IQR]: 77.00–81.00), and patients were predominantly male (61.5%). Table 1 describes the demographic, clinical, and surgical characteristics stratified according to the number of retrieved lymph nodes (< 25 or ≥ 25). Patients’ clinical and demographic characteristics, including age, sex, body mass index, ASA Physical Status classification, comorbidities, Charlson comorbidity index, ECOG performance status, Barthel index, frailty status, nutritional state, calf circumference, hemoglobin, and serum albumin, did not differ between groups.

**Table 1:** Demographic, clinical and surgical characteristics according to the number of retrieved nodes (< or ≥ 25)

	N (%)	≥25 retrieved nodes (n=64)	<25 retrieved nodes (n=58)	p value
Age (median)(IQR)(years)	79.00 (77.00-81.00)	78.50 (77.00-81.00)	79.00 (77.00-82.75)	0.245*
Sex				0.422
Male	75 (61.5)	42 (65.6)	33 (56.9)	
Female	47 (38.5)	22 (34.4)	25 (43.1)	
Body mass index (median)(IQR)(kg/m^2^)	21.80 (20.30-24.68)	21.50 (20.45-24.27)	21.95 (20.30-25.28)	0.533*
ASA classification				0.629
I	0 (0.0)	0 (0.0)	0 (0.0)	
II	17 (13.9)	9 (14.1)	8 (13.8)	
III	99 (81.2)	53 (82.8)	46 (79.3)	
IV	6 (4.9)	2 (3.1)	4 (6.9)	
Comorbidity				
Hypertension	74 (60.6)	39 (60.9)	35 (60.3)	1.000
Diabetes mellitus	23 (18.8)	16 (25.0)	7 (12.1)	0.111
Chronic obstructive pulmonary disease	14 (11.4)	7 (10.9)	7 (12.1)	1.000
Chronic renal disease	14 (11.4)	9 (14.1)	5 (8.6)	0.511
Charlson comorbidity index (median)(IQR)(points)	6.00 (5.00-7.00)	6.00 (5.00-6.00)	6.00 (5.25-6.00)	0.871*
ECOG score				0.476
0	15 (30.6)	9 (30.0)	6 (31.6)	
1	25 (51.0)	17 (56.7)	8 (42.1)	
2	6 (12.3)	2 (6.7)	4 (21.1)	
3	3 (6.1)	2 (6.7)	1 (5.3)	
4	0 (0.0)	0 (0.0)	0 (0.0)	
Barthel index	90.00 (70.00-100.00)	90.00 (88.75-100.00)	85.00 (80.00-100.00)	0.311*
Frailty state				0.904
Robust	2 (7.1)	1 (5.6)	1 (10.0)	
Pre-frailty	17 (60.7)	11 (61.1)	6 (60.0)	
Frailty	9 (32.2)	3 (33.3)	3 (30.0)	
Nutritional state				0.301
Normal	13 (11.1)	7 (12.1)	6 (11.1)	
Risk of malnutrition	57 (48.7)	24 (41.4)	30 (55.6)	
Malnutrition	47 (40.2)	27 (46.6)	18 (33.3)	
Calf circumference	31.75 (29.70-33.23)	31.00 (29.50-33.15)	32.00 (30.00-33.50)	0.460*
Hemoglobin (median)(IQR)(mg/dL)	10.35 (9.00-12.50)	9.80 (8.80-12.00)	9.75 (8.70-12.00)	0.203*
Seric albumin (median)(IQR)(mg/dL)	3.60 (3.20-4.01)	3.50 (3.10-3.80)	3.80 (3.30-4.16)	0.062*
Differentiation				1.000
Differentiated	59 (49.6)	30 (49.2)	29 (50.0)	
Undifferentiated	60 (50.4)	31 (50.8)	29 (50.0)	
Histology				0.942
Intestinal	81 (70.4)	43 (69.4)	38 (71.7)	
Diffuse	22 (19.1)	12 (19.4)	10 (18.9)	
Mixed	12 (10.5)	7 (11.3)	5 (9.4)	
Stage				**0.042**
I	15 (12.2)	5 (7.8)	10 (17.2)	
II	28 (23.0)	11 (17.2)	17 (29.3)	
III	79 (64.8)	48 (75.0)	31 (53.4)	
Tumor location				0.100
Lower	70 (54.7)	32 (50.8)	38 (65.5)	
Middle	34 (26.6)	23 (36.5)	11 (19.0)	
Upper	24 (18.8)	8 (12.7)	9 (15.5)	
Surgical type				**0.035**
Subtotal gastrectomy	71 (58.2)	31 (48.4)	40 (69.0)	
Total gastrectomy	51 (41.8)	33 (51.6)	18 (31.0)	
Surgical approach				0.954
Open	109 (84.5)	55 (85.9)	51 (87.9)	
Laparoscopic	20 (15.5)	9 (14.1)	7 (12.1)	
Adjacent organ resection				1.000
No	117 (95.9)	61 (95.3)	56 (96.6)	
Yes	5 (4.1)	3 (4.7)	2 (3.4)	
Resection margin				1.000
R0	108 (88.5)	57 (89.1)	51 (87.9)	
R+	14 (11.5)	7 (10.9)	7 (12.1)	
Retrieved lymph nodes(median)(IQR)	25.00 (18.00-32.00)	32.00 (28.00-37.25)	17.00 (13.00-21.00)	<0.001*
Metastatic lymph nodes(median)(IQR)	4.00 (0.00-12.00)	7.50 (2.00-12.50)	1.50 (0.00-8.75)	0.002*
Lymph node ratio	0.14 (0.00-0.42)	0.19 (0.05-0.39)	0.09 (0.00-0.49)	0.207*
Neoadjuvant treatment				1.000
No	110 (90.2)	58 (90.6)	52 (89.7)	
Yes	12 (9.8)	6 (9.4)	6 (10.3)	
Adjuvant treatment				0.227
No	69 (56.6)	40 (62.5)	29 (50.0)	
Yes	53 (43.4)	24 (37.5)	29 (50.0)	

Bold values indicate statistically significant p values (p < 0.05)

*p* values were obtained using the Chi-squared test

*p values were obtained using the M t test

Furthermore, no differences were observed between groups regarding tumor histology, differentiation, location, surgical approach, adjacent organ resection, or resection margin. However, tumor stage and type of gastrectomy were statistically different between groups. Neoadjuvant and adjuvant treatments did not differ significantly between groups. In the group with ≥ 25 retrieved lymph nodes, the number of metastatic lymph nodes was significantly higher (median 7.50; IQR: 2.00–12.50) compared with the group with < 25 retrieved lymph nodes (median 1.50; IQR: 0.00–8.75; *p* = 0.002).

Regarding postoperative outcomes, no statistically significant differences were observed between groups in hospital stay duration, intensive care unit stay, need for reintervention, or major complications (Table 2).

**Table 2:** Outcomes according to the number of retrieved nodes (< or ≥ 25)

	*N* (%)	≥ 25 retrieved nodes (*n* = 64)	< 25 retrieved nodes (*n* = 58)	*p* value
Hospital stay (median)(IQR)(days)	7.00 (5.00–11.00)	7.00 (4.00–11.00)	6.00 (5.00-11.75)	0.669*
Intensive care unit stay (median)(IQR)(days)	0.50 (0.00–4.00)	0.00 (0.00–3.00)	1.00 (0.00-5.75)	0.145*
Reintervention				0.619
No	98 (80.3)	53 (82.8)	45 (77.6)	
Yes	24 (19.7)	11 (17.2)	13 (22.4)	
Major complication (Clavien-Dindo ≥ III)				1.000
No	85 (69.7)	45 (70.3)	40 (69.0)	
Yes	37 (30.3)	19 (29.7)	18 (31.0)	

Bold values indicate statistically significant p values (*p* < 0.05)

*p* values were obtained using the Chi-squared test

*p values were obtained using the 2-tailed *t* test

**Fig. 1:**
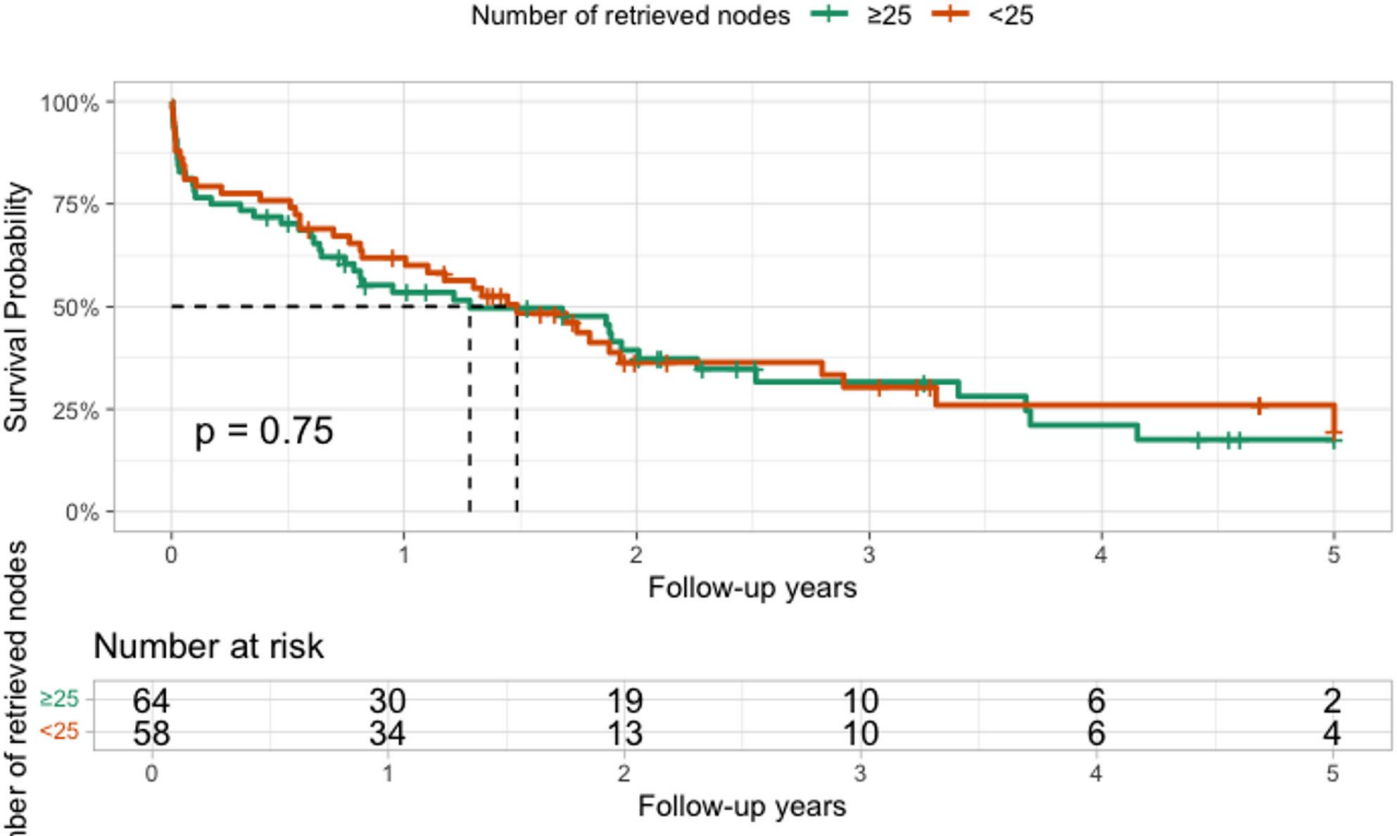
OS for patients older than 75 years comparing the number of retrieved nodes during surgery (< or ≥ 25)

The mean follow-up period for the 122 patients was 17.76 ± 17.31 months (IQR: 4.32–24.12 months). No differences in overall survival (OS) were observed according to the number of retrieved lymph nodes (< 25 vs. ≥25; *p* = 0.75) (Fig. 1).

**Fig. 2:**
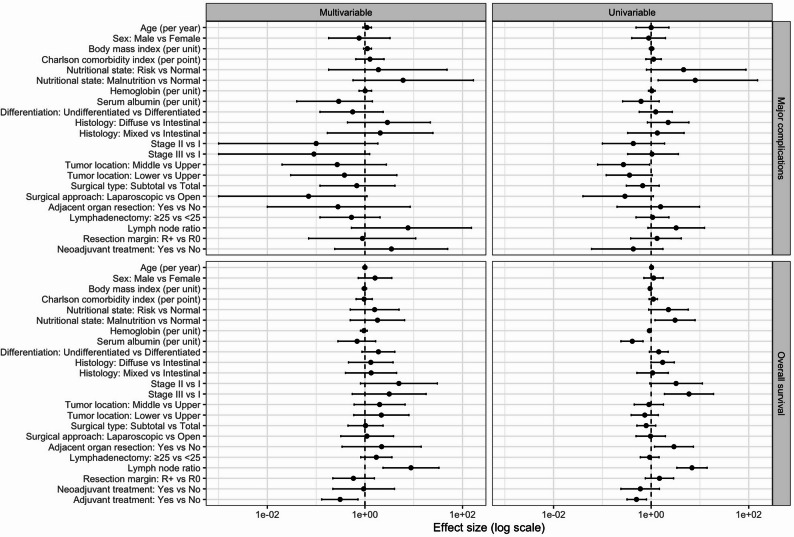
Forest plots non-parsimonious model to predict major complications and overall survival

A multivariable Cox proportional hazards regression analysis (non-parsimonious model) was performed to identify risk factors for OS. The analysis showed that lymph node ratio (HR, 8.79; 95% CI, 2.35–32.85; *p* = 0.001) and adjuvant treatment (HR, 0.31; 95% CI, 0.13–0.72; *p* = 0.006) were independent predictors of OS, whereas the number of retrieved lymph nodes was not independently associated with OS (Table 3; Fig. 2).

**Table 3:** Non-parsimonious model to predict major complications and overall survival

	Major complications (Clavien-Dindo³3)				Overall survival			
	Univariable		Multivariable		Univariable		Multivariable	
	OR (95% CI)	p value	OR (95% CI)	p value	HR (95% CI)	p value	HR (95% CI)	p value
Age	1.00 (0.49-2.31)	0.944	1.10 (0.91-1.36)	0.330	1.02 (0.97-1.07)	0.547	1.00 (0.91-1.09)	0.919
Sex								
Female	Ref		Ref		Ref		Ref	
Male	0.89 (0.40-1.97)	0.762	0.76 (0.18-3.26)	0.709	1.12 (0.71-1.76)	0.627	1.61 (0.73-3.55)	0.236
Body mass index	1.02 (0.92-1.13)	0.716	1.12 (0.94-1.36)	0.215	0.95 (0.89-1.01)	0.097	0.97 (0.88-1.08)	0.586
Charlson comorbidity index	1.12 (0.78-1.61)	0.523	1.27 (0.65-2.46)	0.471	1.11 (0.91-1.35)	0.303	0.96 (0.66-1.41)	0.846
Nutritional state								
Normal	Ref		Ref		Ref		Ref	
Risk of malnutrition	4.62(0.80-87.7)	0.158	1.89 (0.18-48.2)	0.629	2.26 (0.89-5.75)	0.086	1.58 (0.50-5.00)	0.435
Malnutrition	8.00 (1.39-152)	0.055	6.05 (0.57-168)	0.184	**3.09 (1.20-7.94)**	**0.019**	1.81 (0.50-6.50)	0.366
Hemoglobin	1.03 (0.88-1.20)	0.714	1.01 (0.76-1.37)	0.923	**0.92 (0.85-0.99)**	**0.033**	0.95 (0.81-1.11)	0.502
Seric albumin	0.62 (0.26-1.46)	0.283	0.29 (0.04-1.42)	0.142	**0.41 (0.24-0.68)**	**0.001**	0.69 (0.28-1.66)	0.403
Differentiation								
Differentiated	Ref		Ref		Ref		Ref	
Undifferentiated	1.24 (0.57-2.71)	0.594	0.56 (0.12-2.36)	0.431	1.43 (0.92-2.23)	0.116	1.89 (0.87-4.09)	0.107
Histology								
Intestinal	Ref		Ref		Ref		Ref	
Diffuse	2.23 (0.84-5.93)	0.105	2.89 (0.44-21.9)	0.276	1.72 (0.99-2.97)	0.054	1.32 (0.46-3.77)	0.601
Mixed	1.34 (0.33-4.72)	0.657	2.06 (0.17-24.7)	0.556	1.07 (0.51-2.24)	0.864	1.34 (0.40-4.45)	0.630
Stage								
I	Ref		Ref		Ref		Ref	
II	0.43 (0.10-1.88)	0.259	0.10 (0.00-1.84)	0.134	3.24 (0.94-11.20)	0.063	4.97 (0.81-30.58)	0.083
III	1.04 (0.33-3.61)	0.950	0.09 (0.00-1.25)	0.083	** 5.96 (1.87-19.01)**	**0.003**	3.15 (0.55-17.94)	0.196
Tumor location								
Upper	Ref		Ref		Ref		Ref	
Middle	0.27 (0.08-0.93)	0.040	0.27 (0.02-2.74)	0.288	0.90 (0.45-1.79)	0.771	2.00 (0.60-6.65)	0.257
Lower	0.36 (0.12-1.05)	0.061	0.38 (0.03-4.50)	0.447	0.74 (0.39-1.40)	0.353	2.17 (0.59-7.97)	0.243
Surgical type								
Total gastrectomy	Ref		Ref		Ref		Ref	
Subtotal gastrectomy	0.67 (0.31-1.46)	0.313	0.68 (0.12-4.12)	0.659	0.79 (0.51-1.24)	0.307	1.03 (0.45-2.35)	0.945
Surgical approach								
Open	Ref		Ref		Ref		Ref	
Laparoscopic	0.29 (0.04-1.11)	0.113	0.07 (0.00-1.10)	0.102	0.97 (0.49-1.96)	0.941	1.10 (0.32-3.86)	0.878
Adjacent organ resection								
No	Ref		Ref		Ref		Ref	
Yes	1.56 (0.20-9.82)	0.633	0.28 (0.01-8.46)	0.480	**2.93 (1.17-7.36)**	**0.022**	2.20 (0.34-14.21)	0.408
Lymphadenectomy								
<25	Ref		Ref		Ref		Ref	
≥25	1.07 (0.49-2.31)	0.871	0.53 (0.12-2.04)	0.361	0.93 (0.60-1.45)	0.757	1.71 (0.82-3.57)	0.155
Lymph node ratio	3.25 (0.86-12.4)	0.080	7.64 (0.53-153)	0.153	**6.86 (3.36-14.01)**	**<0.001**	**8.79 (2.35-32.85)**	**0.001**
Resection margin								
R0	Ref		Ref		Ref		Ref	
R+	1.32 (0.38-4.14)	0.642	0.89 (0.07-11.0)	0.921	1.48 (0.76-2.89)	0.246	0.58 (0.22-1.56)	0.279
Neoadjuvant treatment								
No	Ref		Ref		Ref		Ref	
Yes	0.43 (0.06-1.74)	0.290	3.49 (0.24-49.7)	0.339	0.60 (0.24-1.48)	0.264	0.94 (0.22-4.04)	0.930
Adjuvant treatment	-	-	-	-				
No					Ref		Ref	
Yes					**0.50 (0.32-0.80)**	**0.003**	**0.31 (0.13-0.72)**	**0.006**

Bold values indicate statistically significant p values (*p* < 0.05)

*Ref* Reference, *HR* Hazard ratio, *OR* Odds ratio

A multivariable logistic regression analysis (non-parsimonious model) was performed to identify risk factors for major complications. No independent predictors of major complications were identified in this model. Univariate analysis showed that middle tumor location was a protective factor for major complications compared with upper tumor location (OR, 0.27; 95% CI, 0.08–0.93; *p* = 0.040) (Table 3; Fig. 2).

A parsimonious multivariable Cox regression analysis was subsequently performed to identify risk factors for OS. Two models were constructed: the first included variables that were statistically significant in the univariate analysis, along with the number of retrieved lymph nodes (< 25 or ≥ 25), which was the primary variable of interest. The second model included lymph node ratio, adjuvant treatment, and the number of retrieved lymph nodes.

**Fig. 3:**
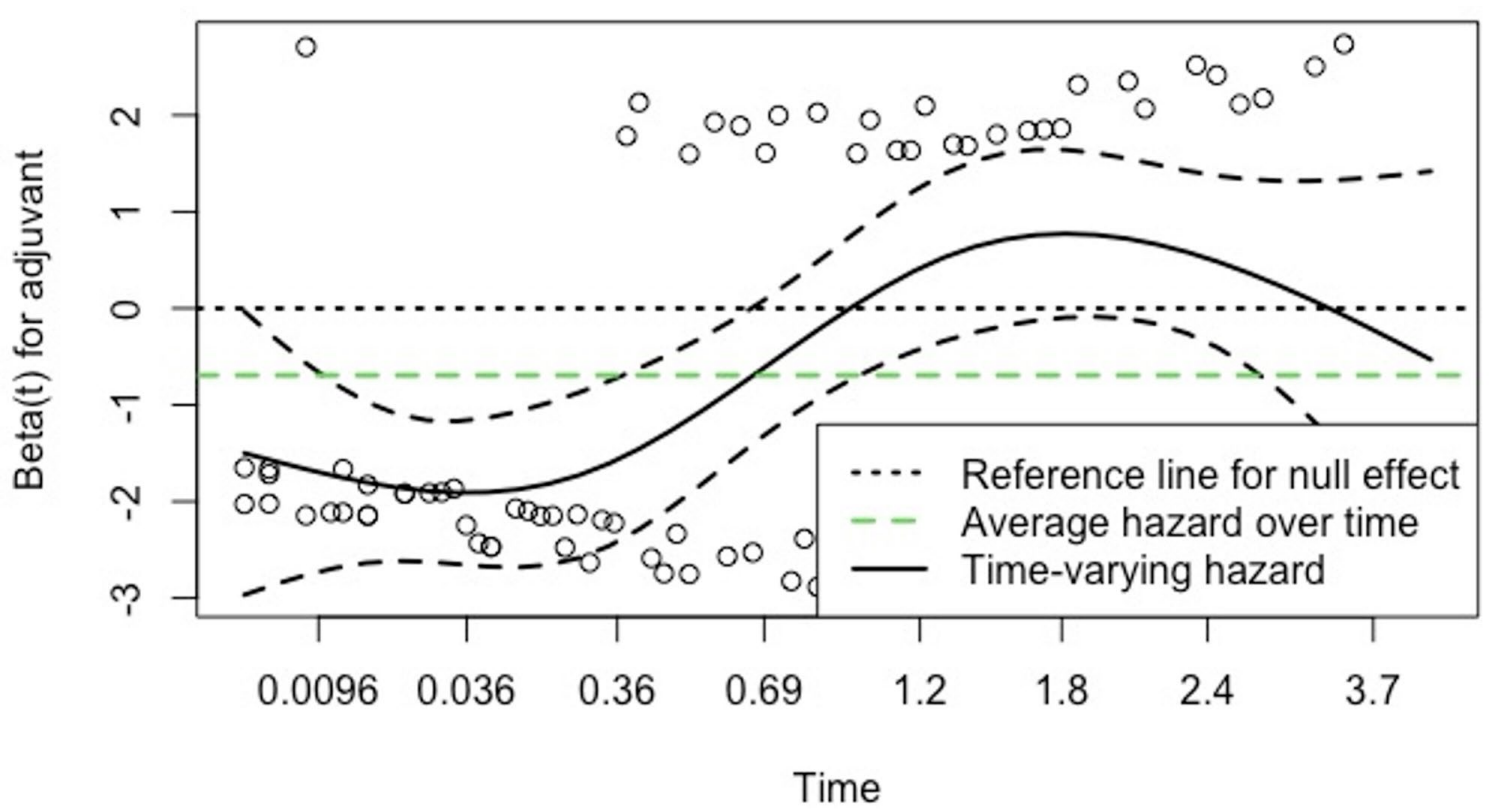
“Adjuvant therapy” covariate effect on the outcome “overall survival” over time

Evaluation of the proportional hazards assumption using Schoenfeld residuals demonstrated that the assumption was not met, as adjuvant treatment showed a time-varying effect. Therefore, a plot was generated to assess the variation of this coefficient over time (Fig. 3). The plot revealed a turning point at approximately 0.36 years, where the beta coefficient changed direction, suggesting a time-varying effect. Accordingly, the follow-up period was divided into two time intervals, and the Cox model was stratified by time.

In the first parsimonious model, lymph node ratio (HR, 5.22; 95% CI, 1.83–14.90; *p* = 0.002), adjacent organ resection (HR, 4.14; 95% CI, 1.01–16.92; *p* = 0.048), and lack of adjuvant treatment before 0.36 years (HR, 13.48; 95% CI, 2.97–61.15; *p* = 0.001) were identified as independent risk factors for OS, whereas the number of retrieved lymph nodes remained non-significant. In the second model, results were consistent, with lymph node ratio (HR, 6.35; 95% CI, 3.08–13.08; *p* = 0.001) and lack of adjuvant treatment before 0.36 years (HR, 27.50; 95% CI, 3.74–202; *p* = 0.001) remaining independent predictors of OS (Table 4). After stratifying follow-up time, the proportional hazards assumption was satisfied for both models (*p* = 0.220 and *p* = 0.310, respectively).

**Table 4:** Parsimonious model to predict major complications and overall survival

	Major complications (Clavien-Dindo 3)		Overall survival (model 1)		Overall survival (model 2)	
	Multivariable		Multivariable		Multivariable	
	OR (95% CI)	*p* value	HR (95% CI)	*p* value	HR (95% CI)	*p* value
Nutritional state					-	-
Normal	Ref		Ref			
Risk of malnutrition	3.82 (0.65-73.3)	0.202	1.70 (0.58-4.97)	0.333		
Malnutrition	7.27 (1.24-139)	0.078	1.43 (0.45-4.58)	0.546		
Hemoglobin	-	-	0.97 (0.86-1.10)	0.652	-	-
Seric albumin	-	-	0.68 (0.33-1.40)	0.295	-	-
Tumor location			-	-	-	-
Upper	Ref					
Middle	0.34 (0.08-1.28)	0.114				
Lower	0.38 (0.11 -1.23)	0.106				
Stage	-	-			-	-
I			Ref			
II			4.42 (0.85-22.93)	0.077		
III			3.76 (0.76-18.73)	0.106		
Lymphadenectomy						
<25	Ref		Ref		Ref	
≥25	0.89 (0.37-2.13)	0.801	1.39 (0.80-2.42)	0.245	0.95 (0.61-1.48)	0.820
Lymph node ratio	-	-	**5.22 (1.83-14.90)**	**0.002**	**6.35 (3.08-13.08)**	**<0.001**
Adjacent organ resection	-	-			-	-
No			Ref			
Yes			**4.14 (1.01-16.92)**	**0.048**		
No adjuvant treatment	-	-				
Before 0.36 years			**13.48 (2.97-61.15)**	**0.001**	**27.50 (3.74-202)**	**0.001**
After 0.36 years			1.63 (0.79-3.35)	0.753	0.98 (0.56-1.73)	0.949

Bold values indicate statistically significant p values (*p* < 0.05)

In the parsimonious logistic regression model assessing factors associated with major complications, no independent predictors were identified (Table 4).

The apparent association between not receiving adjuvant therapy before 0.36 years and worse overall survival should not be interpreted as a causal effect. This finding most likely reflects confounding by early postoperative mortality and severe complications, as patients who died or experienced major morbidity early after surgery were not candidates for adjuvant treatment. Accordingly, the effect of adjuvant therapy disappeared after 0.36 years, which is inconsistent with the established long-term benefit of adjuvant treatment and supports a time-dependent bias rather than a true therapeutic effect. In contrast, lymph node ratio emerged as the most robust and consistent independent predictor of overall survival, remaining statistically significant across all proposed models.

## Discussion

This study analyzed a retrospective cohort of patients aged ≥ 75 years who underwent gastrectomy with curative intent for GC, comparing surgical and oncological outcomes according to the number of retrieved lymph nodes (< 25 vs. ≥25). We did not identify statistically significant differences in overall survival between groups, a finding that is consistent with previous studies, such as that by Passot et al. [22], in which a multicenter cohort of 386 patients aged ≥ 75 years was evaluated according to the number of resected lymph nodes (< 15, 15–25, and > 25), without significant differences in OS among groups. Other observational studies comparing limited (D1) and extended (D2) lymphadenectomy in gastric cancer surgery have also reported no differences in OS, although some have described higher complication rates associated with extended lymphadenectomy [3, 16, 18, 27–30]. To our knowledge, the only study reporting an oncological survival benefit associated with a higher lymph node yield is the population-based cohort published by Brenkman et al. [23]. In that study, patients were also stratified according to the number of retrieved lymph nodes (< 15, 15–25, and > 25), and higher OS rates were observed in groups with greater lymph node retrieval. However, it is important to note that the median number of retrieved lymph nodes in patients aged ≥ 75 years in that cohort was 11 (IQR: 6–18), with only 13% of patients achieving a high lymph node yield, compared with our cohort, in which the median number of retrieved nodes was 25.00 (IQR: 18.00–32.00). This distinction is particularly relevant in older adults, who generally have shorter life expectancy and a higher risk of death from competing causes [31, 32]. As a result, it is unlikely that many older patients will reach the 15-year postoperative follow-up required to observe the long-term survival benefits associated with extended lymphadenectomy [13, 14].

This study also identified lymph node ratio as an independent risk factor for OS. Lymph node ratio is widely regarded as a robust prognostic indicator, as it may reduce the impact of stage migration, and has been consistently associated with OS, disease-free survival, and cancer-specific survival, independently of the total number of examined lymph nodes [33–36]. This prognostic value has been consistently demonstrated in systematic reviews and meta-analyses [37], in which lymph node ratio has been shown to outperform absolute lymph node count in predicting survival outcomes, particularly when lymph node retrieval is heterogeneous. Notably, both the number of metastatic lymph nodes [7.50 (IQR: 2.00–12.50) vs. 1.50 (IQR: 0.00–8.75), *p* = 0.002] and the lymph node ratio [0.19 (IQR: 0.05–0.39) vs. 0.09 (IQR: 0.00–0.49), *p* = 0.207] were higher in the group with ≥ 25 retrieved lymph nodes. In addition, this group exhibited a higher proportion of total gastrectomies and more advanced tumor stages.

Taken together, these findings—along with the similar OS observed between groups—suggest that the intraoperative decision-making process regarding surgical radicality was appropriate and aligned with tumor burden and stage. Because groups in this study were defined according to the number of retrieved lymph nodes rather than surgical technique or planned lymphadenectomy extent, no definitive or high-quality recommendations regarding surgical approach can be derived from these data alone.

Comprehensive evaluation of frailty, functional status, nutritional reserve, sarcopenia, and physiological resilience may provide prognostic information not fully captured by anatomical or pathological variables alone. Future surgical decision-making models in elderly gastric cancer patients should therefore integrate objective functional metrics alongside oncological parameters to better individualize treatment strategies [38, 39].

Several biological and physiological mechanisms may help explain the lack of overall survival benefit associated with higher lymph node retrieval in older patients. Aging is associated with immunosenescence, characterized by impaired antitumor immune surveillance, which may limit the oncological impact of more extensive nodal clearance [40]. In addition, older adults have reduced physiological reserve and increased vulnerability to surgical stress and systemic inflammatory responses, potentially offsetting any marginal oncological advantage gained through extended lymphadenectomy. Finally, competing risks of non-cancer–related mortality are more prominent in this population, which may dilute long-term survival benefits that only become evident after prolonged follow-up. Together, these factors suggest that the balance between oncological radicality and biological resilience differs substantially in older patients compared with younger cohorts.

Although lack of adjuvant therapy has been described as an independent risk factor for overall survival in previous studies [41], the association observed in our analysis is most likely attributable to confounding driven by early postoperative mortality and severe complications, rather than a true causal effect of adjuvant therapy. In this context, non-receipt of adjuvant treatment appears to function as a surrogate marker of postoperative fitness, reflecting underlying frailty, limited physiological reserve, and reduced capacity to tolerate systemic therapy. This interpretation is supported by prior literature demonstrating that frailty indices, functional impairment, and poor treatment compliance are strongly associated with both inability to complete adjuvant therapy and worse survival outcomes in older oncology patients. Accordingly, the disappearance of this effect over time in our analysis is consistent with a selection phenomenon rather than a sustained therapeutic impact.

The rate of major postoperative complications in our cohort was relatively high (30.3%), a finding that is consistent with studies comparing surgical outcomes between high-income and middle-income countries [42]. This rate is comparable to that reported by Rausei et al. [27], who found no significant differences in OS between D1 and D2 lymphadenectomy, but observed a trend toward higher complication rates in the extended lymphadenectomy group, particularly among patients with greater comorbidity burden. In our study, no independent predictors of major complications were identified. Additionally, the observed distribution of differentiated tumors (50.4%), intestinal histological type (69.7%), and lower tumor location (54.7%), which are generally associated with more favorable prognosis, is consistent with previous reports and may suggest that extended lymphadenectomy is not routinely necessary in older populations. Current evidence indicates that advanced age and poor functional status, commonly assessed using the ASA Physical Status classification and ECOG performance status, are important risk factors for postoperative mortality [42]. Although variables such as frailty, functional status, and ECOG score were collected to better characterize our cohort, they were not included in multivariable models due to missing data. The high comorbidity burden observed in this cohort, reflected by a median Charlson comorbidity index of 6.00 (IQR: 5.00–7.00), may further explain the elevated complication rate [27]. As the primary objective of this study was to balance oncological benefit against surgical risk in older patients, these findings are clinically relevant. Postoperative complications and mortality not only have a substantial impact on patients and their families, but also compromise treatment continuity and impose a significant burden on healthcare systems.

This study has several limitations. First, its retrospective design limited the availability of some relevant variables, including surgical technique details, operative time, blood transfusion requirements, and postoperative intensive care unit stay. Inherent to this design, selection bias cannot be excluded, as only patients deemed suitable for gastrectomy with curative intent were included, potentially representing a fitter subgroup of older adults. In addition, comprehensive data on cognitive status, frailty, and socioeconomic conditions were unavailable for many patients. Furthermore, the extent of lymphadenectomy was not standardized and depended on intraoperative findings and surgeon judgment, which may have introduced variability and residual confounding related to disease severity and patient fitness. Another limitation is that cause-specific mortality was not analyzed, and outcomes were assessed using all-cause mortality. Nevertheless, we believe this exploratory analysis provides valuable insight into surgical decision-making in an understudied population for which definitive evidence-based recommendations remain limited.

## Conclusion

Although extended lymphadenectomy allows the resection of a higher number of lymph nodes, improves pathological staging, and has demonstrated oncological benefits in younger populations, evidence supporting its routine use in older adults remains limited. In our cohort, no improvement in overall survival was observed according to the number of retrieved lymph nodes, while a substantial rate of major postoperative complications was identified. Although the number of retrieved lymph nodes does not directly reflect the surgical technique employed, limited lymphadenectomy may represent a reasonable option in patients with early-stage GC or in those whose comorbidities preclude more extensive surgery, particularly in the presence of favorable tumor biology, limited disease burden, and impaired functional or physiological reserve. Conversely, in carefully selected patients with adequate physiological reserve and postoperative support, extended lymphadenectomy may still offer oncological benefit. Therefore, surgical decision-making should be individualized, integrating patient condition, tumor characteristics, and therapeutic goals, including functional status, frailty, and anticipated tolerance to multimodal treatment. Prospective and interventional studies are needed to better define the risk–benefit balance of lymphadenectomy extent in older adult patients with gastric cancer. 

## Data Availability

Data are available on request through the institutional review board of Hospital Universitario Mayor - Méderi. You can contact to request the data at ramirezgiraldocamilo@gmail.com.
